# Rbpj direct regulation of *Atoh7* transcription in the embryonic mouse retina

**DOI:** 10.1038/s41598-018-28420-y

**Published:** 2018-07-05

**Authors:** Joel B. Miesfeld, Myung-soon Moon, Amy N. Riesenberg, Ashley N. Contreras, Rhett A. Kovall, Nadean L. Brown

**Affiliations:** 1 Department of Cell Biology & Human Anatomy, University of California Davis School of Medicine, One Shields Avenue, Davis, CA 95616 USA; 2 Division of Developmental Biology, Cincinnati Children’s Hospital Research Foundation, 3333 Burnet Avenue, Cincinnati, OH 45229 USA; 3 Department of Molecular Genetics, Biochemistry and Microbiology, University of Cincinnati School of Medicine, Cincinnati, OH 45267 USA; 4 Present Address: Department of Biology, University of Cincinnati Blue Ash College, Cincinnati, OH 45236 USA

## Abstract

In vertebrate retinal progenitor cells, the proneural factor *Atoh7* exhibits a dynamic tissue and cellular expression pattern. Although the resulting *Atoh7* retinal lineage contains all seven major cell types, only retinal ganglion cells require *Atoh7* for proper differentiation. Such specificity necessitates complex regulation of *Atoh7* transcription during retina development. The Notch signaling pathway is an evolutionarily conserved suppressor of proneural bHLH factor expression. Previous *in vivo* mouse genetic studies established the cell autonomous suppression of *Atoh7* transcription by *Notch1*, *Rbpj* and *Hes1*. Here we identify four CSL binding sites within the *Atoh7* proximal regulatory region and demonstrate Rbpj protein interaction at these sequences by *in vitro* electromobility shift, calorimetry and luciferase assays and, *in vivo* via colocalization and chromatin immunoprecipitation. We found that Rbpj simultaneously represses *Atoh7 t*ranscription using both Notch-dependent and –independent pathways.

## Introduction

During vertebrate embryonic development, multipotent retinal progenitor cells (RPCs) undergo a prolonged period of differentiation during which six neuronal (retinal ganglion, cone and rod photoreceptor, amacrine, horizontal and bipolar) and one glial (Müller) cell type are produced and assembled into a highly laminated tissue. Multiple signaling pathways and transcription factors, including proneural basic helix-loop-helix (bHLH) transcription factors, regulate retinal cell fate, as reviewed in^1^. Among these factors, *Atoh7* (Atonal homolog 7, *Math5, Ath5*) is dynamically expressed by subsets of embryonic RPCs and required for retinal ganglion cell (RGC) formation^2–6^. Without *Atoh7* function, essentially all RGCs fail to differentiate and adult animals lack optic nerves^3,5,6^. Although the *Atoh7* retinal lineage includes all seven major cell types, gene activity is only required for RGC genesis. To understand how *Atoh7* acts as an RGC competence factor requires deeper understanding of its mode of action as a DNA-binding protein, and the mechanisms tightly regulating its mRNA and protein. Here we focus on particular aspects of *Atoh7* transcriptional regulation.

Previous studies of vertebrate *Atoh7* genomic architecture defined two enhancer regions on the 5′ side of the lone *Atoh7* coding exon, each containing multiple conserved noncoding elements (CNEs)^7–10^. In mice, the distant, shadow enhancer is 9.5 kb and the proximal, primary enhancer is 1.5Kb upstream of the *Atoh7* ATG start codon. The primary enhancer is further subdivided into two CNEs, termed distal and proximal. The distal CNE contains validated Pax6 (paired domain) and Neurog2 (E box) binding sites, through which Pax6 activates transcription, and Neurog2 drives the initial wave of retinal neurogenesis^2,10–12^. However, the activities of these two factors cannot account for the rapid downregulation of *Atoh7* in subsets of RPCs at differentiation.

Given that Notch signaling regulation of bHLH factors occurs widely, including throughout the vertebrate nervous system, we wish to understand how this pathway controls *Atoh7* expression at the molecular level, in the context of retinogenesis. Canonical Notch signaling initiates with a Delta-like or Jagged/Serrate ligand on one cell binding to a Notch receptor on an adjacent cell. This triggers proteolytic cleavage of the receptor to ultimately release its intracellular domain (termed NICD). The NICD complexes with a CSL protein (**C**BF-1, RBPJ-κ, Recombination Signal Binding Protein for immunoglobulin kappa J region) in human/mouse, **S**u(H) (Suppressor of Hairless) in *Drosophila*, **L**ag-1 (*lin12* and *glp-1* phenotype) in *C. elegans*) and the Maml (Mastermind-like) co-activating factor. This protein complex activates downstream target gene transcription by binding to a variety of CSL sites within noncoding DNA^13–16^. Well-characterized effector genes of the Notch pathway include the *Hes* (Hairy/Enhancer of Split (E(Spl)) and *Hey* gene families, which encode transcriptional repressor proteins^17–19^. Intriguingly, during *Drosophila* retinal development, Notch signaling reiteratively regulates *Atonal* transcription^20,21^. In undifferentiated cells anterior to the eye disc morphogenetic furrow (a moving differentiation boundary), Notch signaling activates *Atonal* in a continuous stripe of cells. But more posteriorly, the Notch complex, via E(Spl) activity, suppresses *Atonal* expression in cells adopting non-R8 photoreceptor fates. This latter activity embodies the classic neurogenic role of Notch signaling. Importantly, these phases of *Atonal* regulation are separated in both time and space, and utilize distinct *Atonal* enhancers, as reviewed in^22^.

Predicted CSL binding sites are interspersed throughout metazoan genomes, but it is challenging to know which are *in vivo* targets. However, the probability of any gene being a direct target is enhanced when its expression is affected by Notch pathway loss- and gain-of-function mutants. Previous examinations of mouse retinal mutants showed that loss of *Notch1*, *Rbpj* or *Hes1* derepresses *Atoh7* mRNA expression and RGC neurogenesis^23–29^. By contrast, overexpression of activated Notch1 (NICD1) stimulates proliferation, thereby blocking *Atoh7*^*LacZ*^ expression and RGC formation^26,30^. Yet, the mechanism(s) underlying Notch pathway regulation of *Atoh7* remain unresolved.

A classical view of Notch regulation of bHLH factors holds that an activated Notch protein complex transcriptionally activates *Hes1*, which in turn would repress *Atoh7* transcription. But, other models are possible, including Rbpj direct regulation of *Atoh7*, either in a corepressor complex (repression), or in a Notch complex (activation)^21,31,32^. However, all genetic data consistently support Rbpj suppression of *Atoh7* transcription. In this study, we characterize four CSL binding sites situated in *Atoh7* 5′ regulatory DNA. We found that at the peak of *Atoh7* retinal expression, Rbpj occupies one CSL site, R3 within the distal primary enhancer, and this binding site mediates transcriptional repression. We also discovered that simultaneous loss of all four binding sites reduced *Atoh7* transcription, suggesting a distinct mode of Rbpj regulation, consistent with locus priming for efficient transcriptional regulation by other factors^33^. Importantly, both Rbpj-mediated repression at site R3, and activation via multiple sites are Notch-independent, although Notch1/3 signaling has a measurable impact on *Atoh7* mRNA levels. Overall our data are suggestive of three separate, yet simultaneous modes of Rbpj regulation: locus priming, direct repression and canonical Notch pathway suppression. We propose that integrated regulatory inputs are important for precise control of *Atoh7* pulsatile expression in the developing retina.

## Results

### Predicted Rbpj binding sites in *Atoh7* regulatory DNA

**Figure 1:**
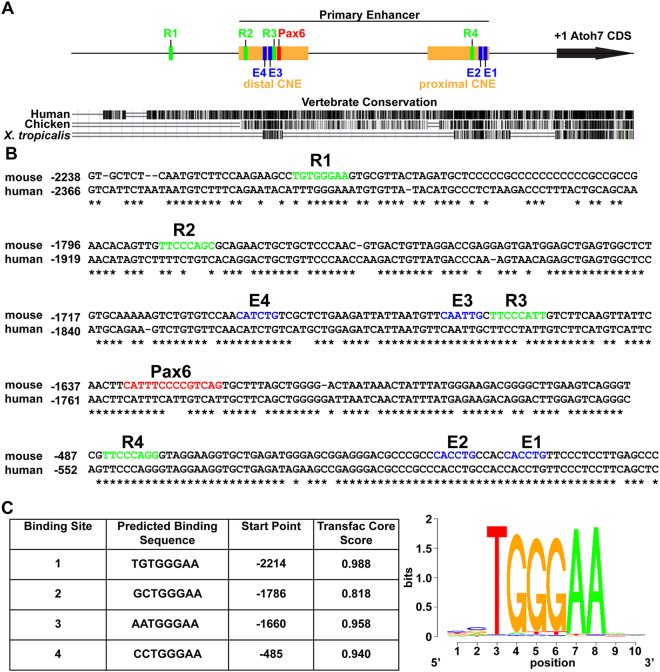
Predicted Rbpj CSL binding sites in mouse *Atoh7* 5′ regulatory DNA. (**A**) Diagram of the primary enhancer and the distal and proximal CNEs, indicating the position of four predicted Rbpj binding sites (R1–R4, green boxes), a previously characterized Pax6 binding site (red box) and four E-boxes binding sites (E1–E4, blue boxes). UCSC Genome browser view of a 3 kb 5′ noncoding region of mouse *Atoh7* (+1 = A of ATG start codon) using mm10 assembly and vertebrate evolutionary conservation tracks, with mouse as the reference genome. (**B**) ClustalW alignments of mouse and human *Atoh7* genomic sequence, with asterisks indicating nucleotide identity diagrammed in (**A**,**C**) The four predicted Rbpj binding site sequences, identified by the Transfac CSL consensus matrix.

The goal of this study was to identify and test putative Rbpj consensus (CSL) binding sites relative to *Atoh7* transcriptional activity. Because the *Atoh7* primary enhancer recapitulates endogenous mRNA expression in frog, chick, zebrafish and mouse transgenic studies, and contains two CNEs, it was the strongest candidate to contain CSL binding sites^8,10,11,34^. However, we used the CSL consensus TRANSFAC MATCH algorithm (5′-[C/T]GTG[G/T]GAA-3′) to perform an unbiased search of 6 Kb of noncoding DNA surrounding the *Atoh7* coding exon^13–15^. Four putative CSL sites (termed R1–R4) were identified; all positioned 5′ to the ATG codon (Fig. 1A). Three of these sites (R2–R4) reside in the primary enhancer CNEs, while the other site (R1) is 300 bp upstream of the primary enhancer (Fig. 1A,B). Of the four sites, R1, R3, and R4 are highly conserved between mouse and human (Fig. 1B), with only R3 and R4 having analogous sequences in the chick and frog *Atoh7*/*Ath5* locus (not shown). Interestingly, site R3 is situated in between previously characterized Pax6 and Neurog2 (E4/E3) binding sites (Fig. 1A,B)^8,10,11^. Site R4 lies within the proximal CNE, very close to the TATAA box and two highly conserved Ebox binding sites (Fig. 1A,B). In the adult frog retina, this CNE is critical for maintaining *Atoh7/Ath5* expression in the ciliary marginal zone^8^.

### Rbpj and Atoh7 antibody validation and protein colocalization

**Figure 2:**
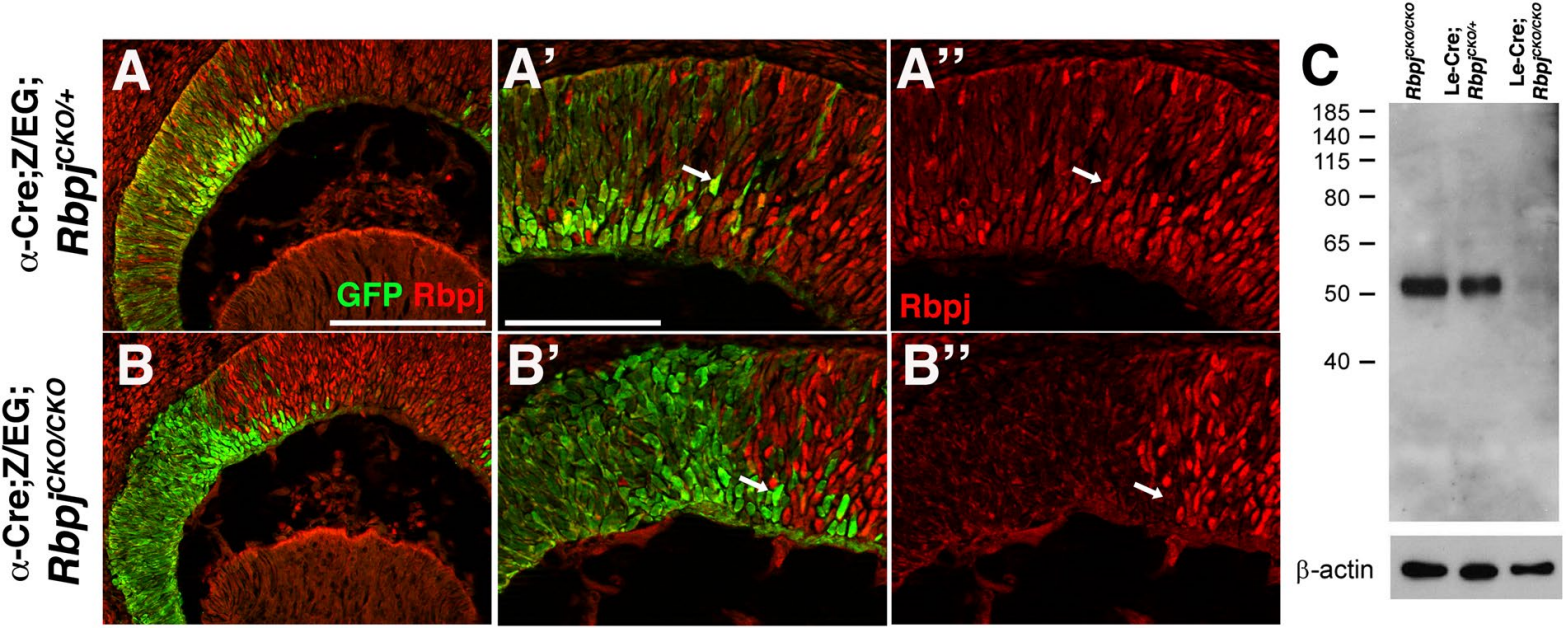
Rbpj antibody specificity. (**A**,**B**) GFP-positive cells (green) indicate the E13.5 α-Cre retinal lineage. **A-A”**) Anti-Rbpj labeling of α-Cre;Z/EG;*Rbpj*^CKO/+^ control cryosections shows ubiquitous expression of the nuclear Rbpj protein (red), including within GFP+ cells (arrow). (**B**-**B”**) Cell autonomous loss of Rbpj expression (red) in conditionally mutant α-Cre;Z/EG;*Rbpj*^CKO/CKO^ cells (arrow). n = 3 biologic replicates embryos/genotype. (**C**) Western blot of E14.5 lens protein extracts, collected from *Rbpj*^CKO/CKO^, Le-Cre;*Rbpj*^CKO/+^ and Le-Cre;*Rbpj*^CKO/CKO^ embryos, respectively. A single band of 56 kDa, the predicted size of Rbpj, is absent from mutant lenses, with β-actin loading control at bottom. The top panel is the uncropped blot ECL autorad exposure, which was stripped and reprobed with Actin and Jagged1 loading controls (see Supplemental Fig S3). (**A**-**B”**) apical is up. Bar in A for A,B = 20μm; in A’-for A’ to B” = 40 μm. White arrows point to GFP+ cells expressing Rbpj in controls (**A-A”**), but not in GFP+;*Rbpj* mutant cells (**B**-**B”**).

Both commercial and academic laboratory-made antibodies have been generated for detecting mammalian Rbpj or Atoh7 proteins. In a separate project, we rigorously tested a polyclonal antibody with high specificity for human and mouse Atoh7 proteins^35^. Previously, a rat anti-Rbpj antibody was used in our exploration of Notch signaling during mouse lens development^36,37^. Here we further confirmed the specificity of this monoclonal antibody, during mouse retinogenesis, and evaluated its usefulness in biochemical assays (Fig. 2). We immunolabeled E13.5 retinal cryosections from α-Cre;Z/EG;*Rbpj*^*CK*^*°*^*/*+^ control and α-Cre;Z/EG;*Rbpj*^*CKO/CKO*^ mutant embryos (Fig. 2A,B). The α-Cre transgene induces Cre-mediated recombination in the distal retina^38^ and Cre-mediated recombination is permanently marked by GFP expression from the Z/EG transgene^39^. In α-Cre;Z/EG;*Rbpj*^CKO/+^ controls, Rbpj protein is ubiquitous, including within the Cre lineage (GFP+ cells) (Fig. 2A). However, the same α-Cre cells lacking *Rbpj* showed a cell autonomous loss of protein expression (Fig. 2B). We also used this antibody for Western blotting, using denatured protein extracts from genotyped and pooled embryonic lenses, including from *Rbpj* conditional mutants^40,41^. A single immunoreactive band of 56 kDa was recognized in the wild type and heterozygous western lanes, but completely missing from homozygous mutant lens extracts (Fig. 2C).

**Figure 3:**
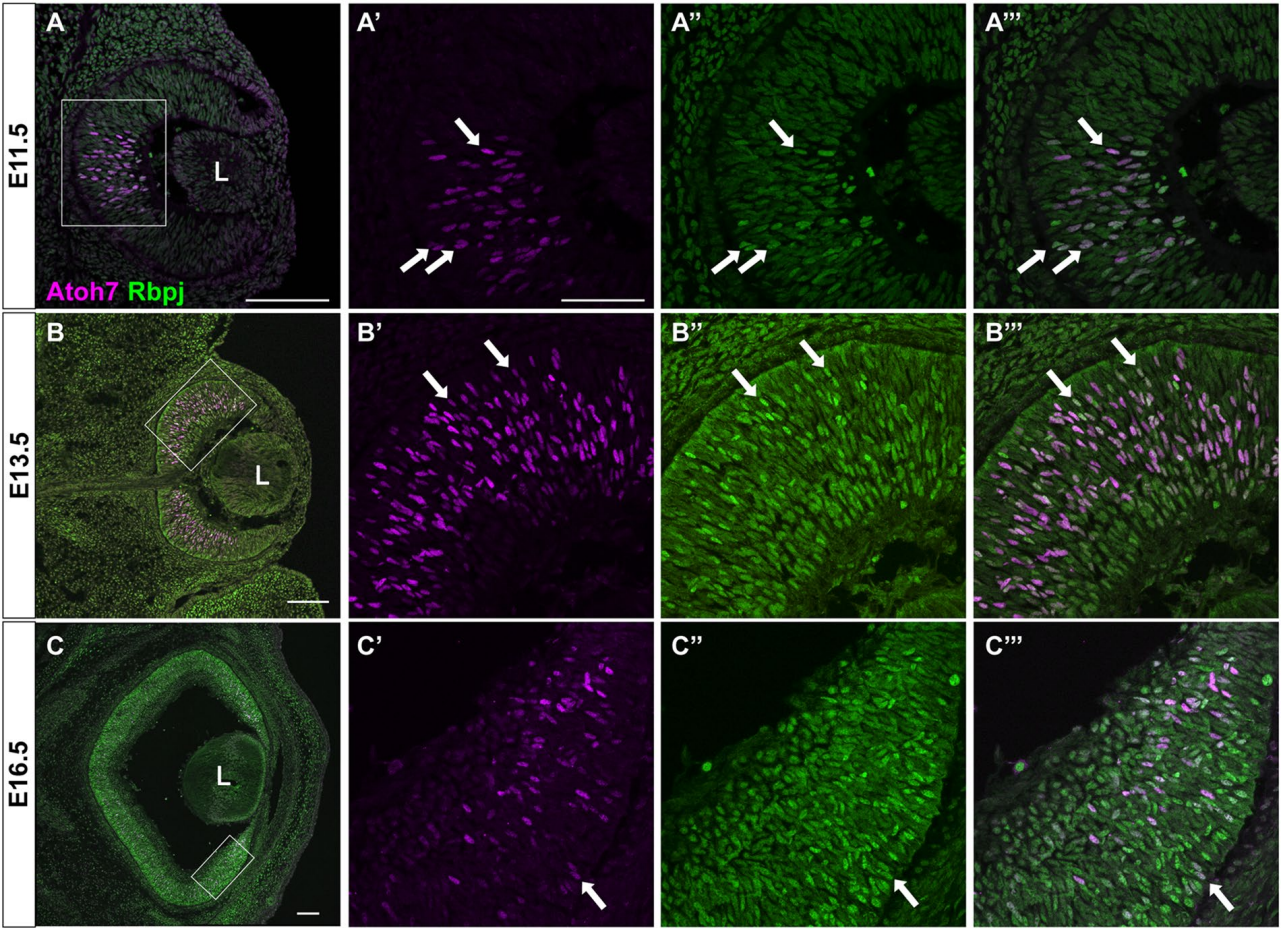
Colocalization of Rbpj and Atoh7 proteins during retinal neurogenesis. (**A**–**C**) Double antibody labeling of retinal sections highlights essentially complete nuclear co-localization. The boxed area in each panel is shown at higher magnification to the right. (**A**-**A’”**) At E11.5 Atoh7 protein is restricted to a subset of central RPCs (**A’**). (**B**-**B’”**) By E13.5 the initial wave of neurogenesis has reached the periphery and Atoh7 is expressed more broadly throughout the apical neuroblast layer. (**C**-**C’”**) Consistent with mRNA expression studies, Atoh7+ cells are localized to the peripheral retina (**C’**). Panel C is a composite stitched together from 4 overlapping 10× image fields. Colocalization of Rbpj (green) and Atoh7 (purple) is shown as white. Rostral is up in all panels. L = lens; Bar in A,C,E = 100 μm; in B,D,F = 50 μm. In (**A’**-**C”’**) white arrows point to Atoh7+ cells that also contain high levels of Rbpj.

Next, we compared Atoh7 and Rbpj protein expression during *in vivo* embryonic retinal development. The spatiotemporal expression pattern of Atoh7 protein is highly dynamic, restricted to the central optic cup at E11.5 (Fig. 3A), but expressed by a broader group of E13.5 RPCs (Fig. 3B), and then confined to the periphery by E16.5 (Fig. 3C). Because Rbpj expression is ubiquitous, complete coexpression was expected. However, we noted that at every age almost all Atoh7+ cells exhibit brighter Rbpj expression than the Atoh7-negative cells. This is suggestive of differential Rbpj protein expression, although immunohistochemistry is a nonquantitative technique. To rule out non-specific secondary antibody cross-reactivity, we performed control experiments on retinal sections that were incubated with only one primary antibody (rabbit anti-Atoh7 or rat anti-Rbpj), prior to simultaneous application of both secondary antibodies (Fig. S1).

### Rbpj binding to *Atoh7* 5′ regulatory DNA *in vitro* and *in vivo*

**Figure 4:**
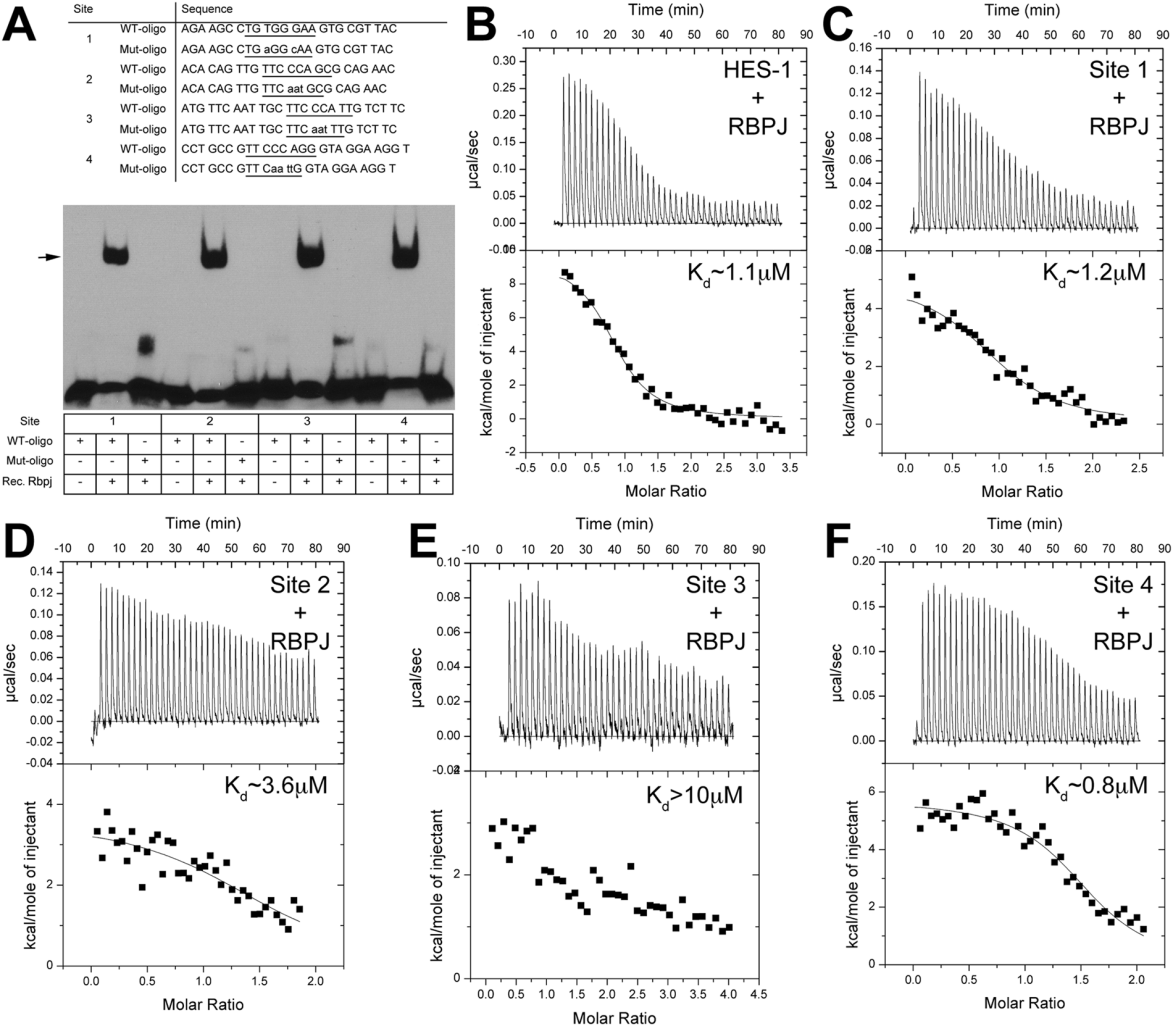
*In vitro* binding of Rbpj to CSL consensus sites in *Atoh7* 5′ DNA. (**A**) Oligonucleotides with CSL consensus sites (underlined) identified in 5′*Atoh7* noncoding DNA, synthesized and used for testing Rbpj protein-DNA binding by EMSA (WT, wild type; Mut, mutated). Lower case indicates a mutated nucleotide. Recombinant Rbpj protein and biotin-labeled oligonucleotide complexes undergo specific mobility shifts (arrow). See uncropped autorad exposure in Supplemental Fig. 3. n = 4 independent assays. (**B**–**F**) Representative thermograms (raw heat signal and nonlinear least squares fit to the integrated data) for *Hes1* CSL consensus site^43^ (positive control in (**B**) and *Atoh7* CSL sites R1–R4, all shown with their dissociation constants (K_d_).

For initial assessment of Rbpj binding to the four putative CSL sites, we conducted *in vitro* electromobility shift assays (EMSA), using bacterially expressed and purified mouse Rbpj protein (residues 53–474), previously shown to bind DNA^42^. Rbpj protein was incubated with biotin-labeled double stranded oligonucleotides, in which the putative binding site is centrally located (Fig. 4A, Supplemental Table S4). We noted that all four sites shifted upon incubation with Rbpj protein (Fig. 4A). When key nucleotides within each CSL site were mutated, based on previous CSL-DNA structural studies, binding was abolished. We conclude that Rbpj protein specifically binds to each CSL binding site *in vitro*.

We also performed isothermal titration calorimetry (ITC) to quantitate binding, using purified Rbpj with the oligomeric DNA duplexes that correspond to the four putative CSL binding sites (Fig. 4B–F and Supplemental Table S5). As positive and negative controls, we tested Rbpj with the CSL binding site from the *Hes1* proximal promoter element (GTTAC**TGTGGGAA**AGAAAG) and the non-specific sequence (GCTACTCATACCTAGAACG), respectively, and detected binding from the Hes1 site (~1 μM K_d_), but did not detect binding from the non-specific site (data not shown). The results showed Rbpj bound to three of the four putative CSL sites with comparable affinity to the well-characterized Hes1 site (Fig. 4B)^43^. However, we found that one consensus site, R3, displayed a lower binding affinity (Fig. 4E). While we were unable to separate any potential effects of nucleotide variation and flanking sequence, interestingly, R3 lies in between validated Pax6 and Neurog2 binding sites^10,11^.

**Figure 5:**
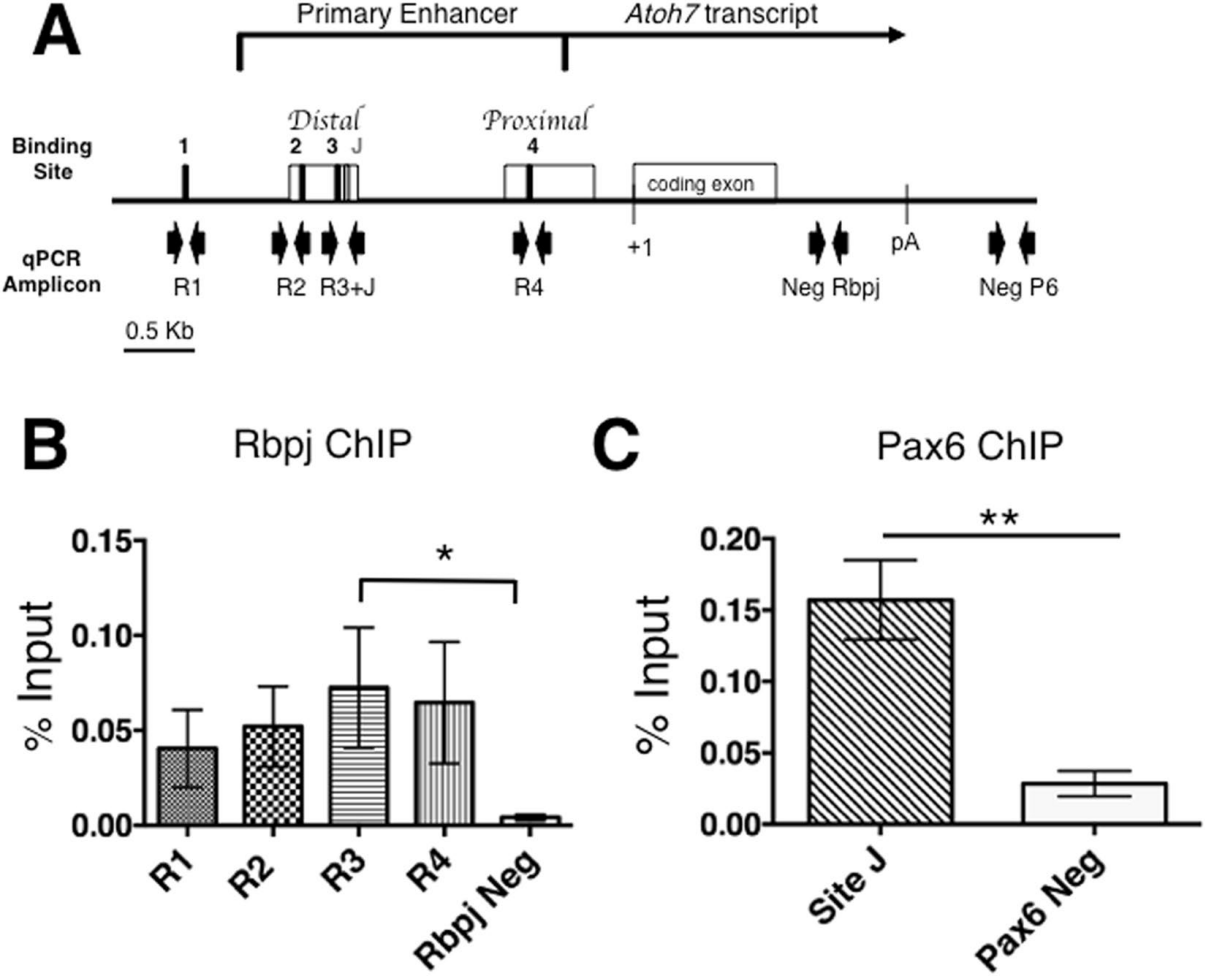
*In vivo* occupancy of Rbpj at *Atoh7* locus in E14.5 retinal chromatin. (**A**) Diagram of mouse *Atoh7* genomic locus indicating CSL binding sites and qPCR amplicons evaluated, with Pax6 site J site serving as positive control. (**B**,**C**). Real-time PCR analyses of DNA fragments amplified after Rbpj or Pax6 ChIP, displayed as the mean ± SEM of three biologic replicate assays, performed in PCR duplicate, with the preimmune IgG values subtracted. Only Rbpj site R3 is significantly enriched over negative control. *p ≤ 0.05; **p ≤ 0.01.

Next, we wished to determine which CSL consensus sites are occupied by Rbpj during *in vivo* retinal development. Previously we demonstrated that Pax6 occupies consensus site J in the human ATOH7 gene, using chromatin from Ad12Her10 retinal cell line^10^. As a positive control, we performed Pax6 ChIP in parallel here, using mouse E14.5 retinal chromatin. This age was selected because it is the peak of *Atoh7* expression^44^. Dissected retinas from individual embryonic litters were pooled and frozen en masse (n = 3 litters). After lysis, cross-linking, quantification and sheering, chromatin was immunoprecipitated with rat anti-Rbpj, rabbit anti-Pax6 or relevant IgG controls (see Methods). The antibody-bound chromatin complexes were purified, crosslinks reversed, and the isolated genomic DNA used as a template for real-time PCR. Each primer set amplified an amplicon specific for each putative binding site (Fig. 5A, Supplemental Table S4). As expected, Pax6 occupies site J in the *Atoh7* primary enhancer (Fig. 5C). Although there was measurable occupancy of Rbpj at all four sites (R1–R4) relative to 3′UTR, only site R3 shows statistically significant enrichment (Fig. 5B). We conclude that Rbpj directly regulates *Atoh7* transcription, via site R3. Although occupancy of the other three sites at E14.5 was not statistically significant, we hypothesized there could be more robust enrichment at other developmental ages, or that Rbpj simultaneous occupancy of multiple sites might contribute to a local *Atoh7* chromatin configuration^33^.

### Rbpj differentially regulates *Atoh7* transcription

**Figure 6:**
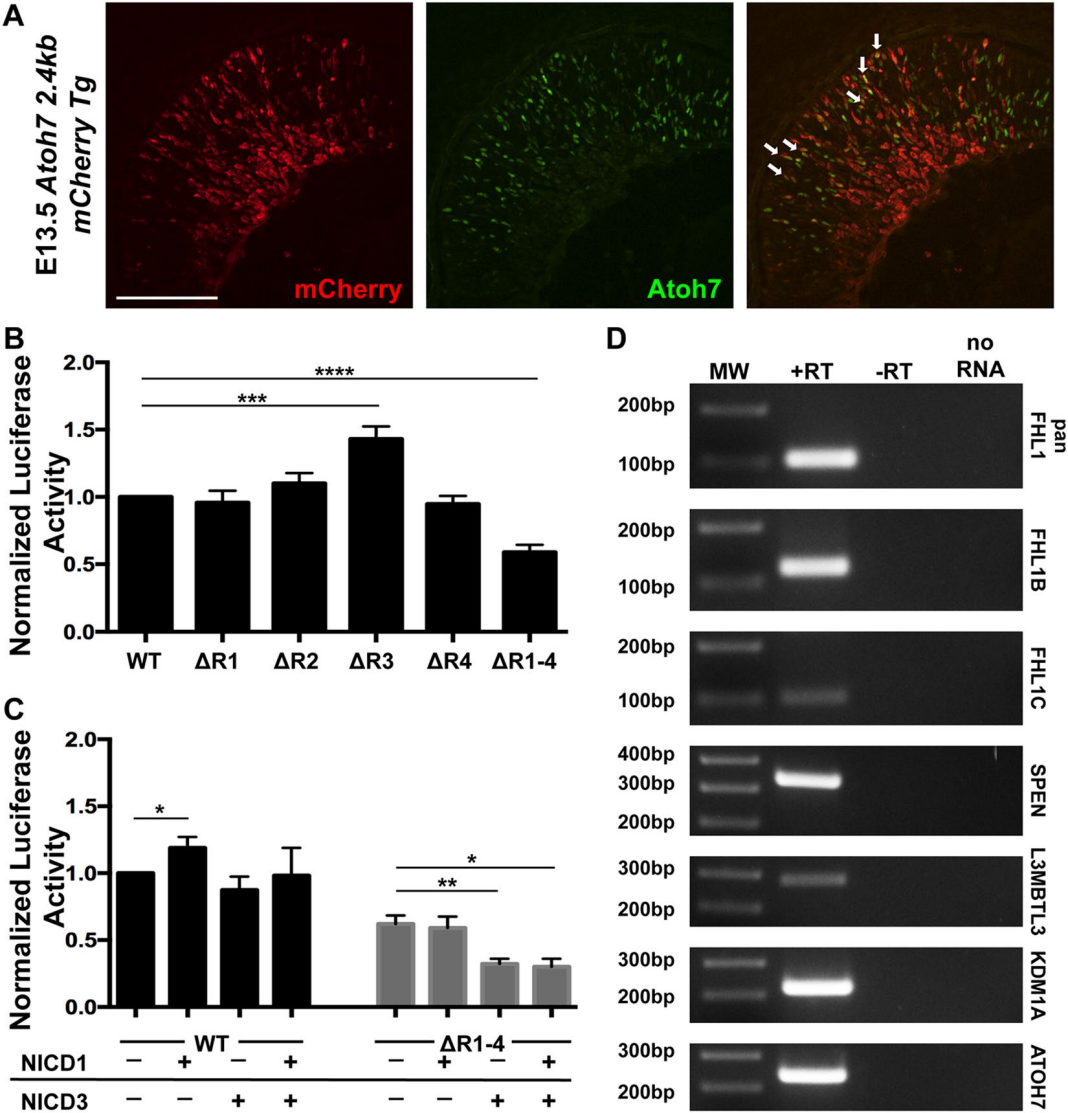
Differential Notch-mediated regulation of *Atoh7* transcription. (**A**) Colocalization of transient mCherry transgenic expression, driven by a 2.4 kb mouse *Atoh7* enhancer, to endogenous Atoh7 protein in the E13.5 retina (White arrows point to colabeled cells). Both cytoplasmic and nuclear Cherry expression are seen, presumably due to the inefficiency of a synthetic nuclear localization sequence and reporter antibody sensitivity, which detected both nascent Cherry protein in the cytoplasm and its accumulation in the nucleus. Scale bar = 100 μm (**B**) Comparison of mouse *Atoh7* transcriptional activity in HEK293T cells following single versus quadruple CSL site mutation. Transcriptional activity of individual CSL site mutants, and quadruple mutant within *Atoh7*–2.6Kb luciferase/pGL2 construct. Only site R3 in the distal CNE is required to suppress *Atoh7* transcription. However, loss of sites R1–4 caused significant downregulation of *Atoh7* transcription. n = 9 biological replicates (each performed in technical triplicate). (**C**) Cotransfection of activated NICD1, NICD3, or both constructs with *Atoh7* wild type or RΔ1–R4 mutant luciferase constructs. n ≥ 3 biological replicates (each in technical triplicate). All luciferase experiments were normalized to a co-transfected Renilla control. A two-tailed, unpaired t-test with equal standard deviation and Gaussian distribution was used to determine p-value. (**D**) RT-PCR analysis of E13.5 retinal cDNA showing expression of all four co-repressor gene mRNAs. Distinct PCR primers for the *Fhl1* (*KyoT*) gene were used that amplified an exon common to all splice products (pan *Fhl*1), and the specific splice variants *Fhl1b* (*KyoT3*) and *Fhl1c* (*KyoT2*) that uniquely contain the Rbpj-interaction domain^78^. All PCR reactions were run on a single gel, uncropped image provided in Supplemental Fig. S3. *p ≤ 0.05; **p ≤ 0.01; ***p ≤ 0.001; ****p < 0.0001 and Figure S2 ***p ≤ 0.001; ****p < 0.0001.

Although Rbpj binds to all four CSL sites *in vitro* and at least site R3 *in vivo*, it is unclear if it does so via the Notch complex (activation), or a corepressor complex (repression). To address these possibilities formally we used luciferase reporter assays to measure the activity and requirement for each site individually, versus simultaneous mutation of all 4 sites, within a previously identified primary gene *Atoh7* enhancer^7,10^. However, first we compared *Atoh7* primary gene enhancer expression to that of the endogenous protein, which had not been previously reported^8,10,11,34,45,46^. For this we created a transgenic construct with 2.4Kb of mouse *Atoh7* 5′ DNA joined to a minimal human β-globin promoter (BG) and red fluorescent monomeric Cherry reporter (mCherry)^47^. We then used antibody colabeling to evaluate mCherry expression in retinal cryosections from E13.5 transient transgenic embryos. We found that endogenous Atoh7 significantly overlaps with Cherry+ cells (Fig. 6A) in the proliferative neuroblast layer, but not in the differentiated ganglion cell layer (GCL). This difference can be attributed to the greater stability of the fluorophore protein compared to Atoh7. Coexpression here was roughly equivalent to what was previously reported for another mouse transgenic line in which the human *ATOH7* shadow enhancer drives BG-mCherry expression^7,35^.

Next, we performed luciferase assays in both HEK293T kidney-derived and AD12Her10 retinal-derived human cell lines, under identical conditions^48,49^. This strategy was chosen because our previous study of Pax6 regulation of *Atoh7* transcription suggested that retinal-specific context influences assay output^10^. Yet we found no differences between these cell lines, although HEK293T cells endogenously express *RBPJ*, but not *ATOH7*^50,51^, whereas AD12Her10 cells express both genes (Supplemental Fig. S2). Individual CSL site mutations (ΔR1, ΔR2, or ΔR4) did not affect transcriptional output, relative to wild type (Fig. 6A). By contrast, our mutation of CSL site R3 (ΔR3) derepressed luciferase activity over wild type levels, in both HEK293T and AD12Her10 cells, in the absence of exogenous Notch intracellular domains (Fig. 6A, Supplemental Fig. S2). We concluded that this particular binding site normally represses *Atoh7* transcription. By contrast, when all four sites (ΔR1–4) were simultaneously mutated, there was a significant decrease in *Atoh7* transcriptional levels (Fig. 6B, Supplemental Fig. 2). This is suggestive of coordinated, Rbpj-mediated transcriptional activation, among multiple (possibly all four) binding sites.

Rbpj occupancy of site R3 represses *Atoh7*, presumably reflecting corepressor complex activity. However, the coordinated enhancement via multiple sites might be attributable to direct regulation of *Atoh7* transcription, via a Notch complex. Therefore, we tested for Notch-dependence by coexpressing the intracellular domains of Notch1 (NICD1) or Notch3 (NICD3) in our luciferase assays. Each receptor alone, as well as in combination, was previously shown to be required *in vivo* for particular aspects of *Atoh7* expression^26^. Plasmids containing the NICD1, NICD3, or a mixture of the two, were cotransfected with either the *Atoh7* wild type or ΔR1–R4 mutant luciferase constructs^52,53^. Increasing NICD1 levels stimulated *Atoh7* transcription but had no impact on the ΔR1–R4 mutant (Fig. 6B). NICD3 or NICD1 + NICD3 coexpression did not affect wild type *Atoh7* activity, but further suppressed transcription in the ΔR1-R4 mutant (Fig. 6B). We interpret these outcomes to mean that multiple CSL binding sites help maintain *Atoh7* basal level transcription. The elevated luciferase activity seen after NICD1 overexpression might represent Notch-dependent regulation of sites R1, R2, and R4, or an ectopic effect of NICD1 acting as a Rbpj sink, de-repressing R3 similar to R3 mutagenesis. In addition, Notch-mediated regulation of *Atoh7* must utilize other noncoding sequences based on the decrease of transcriptional activity with NICD3 overexpression, potentially through Hes consensus N-boxes^16,43,54,55^.

Multiple mammalian “co-repressor” genes encode proteins that interact with Rbpj via CSL binding sites^56^. These genes are unrelated at the primary sequence level, but predicted to link Rbpj to HDAC machinery, and include *Smrt/Ncor2*, *Spen/Mint/Sharp*, *Fhl1b//KyoT2*, *Rita*, *Skip*, *L3mbtl3* and *Kdm1a/Lsd1*^57–59^. Interestingly, Kdm1a/Lsd1 is expressed in the retina from E17-P15, and its pharmacologic inhibition in retinal explants induced an upregulation of bHLH factor expression^60^. Because nothing further is known about the retinal expression other Rbpj co-repressor genes, we used RT-PCR to test for transcription of four genes (*Spen; Fhl1b; L3mbtl3; Kdm1a*) in the E13.5 mouse retina (Fig. 6C). The *Fhll/KyoT* gene encodes multiple splice products, so we assayed for one exon common to all splice variants, as well as specific *Fhl1b* and *Fhl1c* exons that uniquely contain Rbpj-interaction domains^61–63^. We found that all of these co-repressor mRNAs are expressed during embryonic retinal neurogenesis (Fig. 6C). Given that at least four of seven putative co-repressors are present in the mammalian retina, elucidation of their specific mechanisms of action will require in-depth, future studies.

## Discussion

Previous studies of Notch signaling in the vertebrate retina genetically linked *Rbpj* activity to *Atoh7* expression and retinal ganglion cell differentiation^24–29^. Notch activity normally suppresses both *Atoh7* and RGC neurogenesis, but the molecular mechanisms for this remain unresolved. Here, we explored the possibility that Rbpj can directly regulate *Atoh7* transcription.

Bioinformatic analysis of noncoding sequences surrounding the mouse *Atoh7* gene identified four putative Rbpj-CSL consensus binding sites. While a CSL consensus sequence is useful for predicting Rbpj target genes^13–15^, it is not the only nucleotide motif this protein can bind^16,43^. One particular binding site arrangement, called SPS (Su(H)-paired site), consists of two CSL binding sites in a head to head configuration separated by ~16 nucleotides^64^. These SPS elements are occupied by dimeric NICD/Rbpj complexes, to regulate transcription^65–68^. Although the *Atoh7* upstream CSL binding sites lack a canonical SPS arrangement, there remains some possibility for such a mechanism, since a cryptic CSL element in the *Hes5* promoter acts in a dimeric SPS complex^65^.

Among the CSL sites analyzed here for the embryonic retina, R3 stands out as unique. Counterintuitively, CSL has the weakest affinity for R3 *in vitro*, but was the only site that ChIPped CSL *in vivo* with statistical significance. Similar phenomenon has been observed for Su(H), the fly CSL ortholog, binding of the *sparkled* (*spa*) enhancer in *Drosophila*, whereby the low affinity Su(H) sites are critical for proper gene expression and patterning driven by *spa*^69^. The location of R3 within the distal CNE is flanked by a Neurog2-dependent Ebox (30 bp upstream) and a Pax6 binding site (20 bp downstream). Such spacing allows one or two helical turns to separate each of these sites. In the developing pancreas, this same distance is permissive for Rbpj physical interaction with the bHLH transcription factor Ptf1a^70^. Interestingly, Ptf1a is also expressed in the developing retina and influences neurogenesis of several retinal cell types^71–73^. In *Drosophila* and *Xenopus* there are other examples of CSL/Rbpj protein interactions with bHLH factors that activate transcription^66,74,75^. Hence, we cannot discount the possibility that Neurog2 (or another bHLH factor) may physically interact with Rbpj. Alternatively, Rbpj occupancy of site R3 might affect local chromatin architecture, thereby displacing activating factors. Because we detected both Rbpj and Pax6 occupancy in E14.5 retinal chromatin (within the same preparation), a mutually exclusive binding mechanism would seem implausible. One caveat was the use of whole retina chromatin, which could obscure distinct configurations of transcription factor binding at the *Atoh7* primary enhancer, among a heterogenic population of retinal cells. For example, in mitotically-active RPCs that do not express *Atoh7*, Rbpj could act as a repressor at site R3. When these cells enter their terminal mitosis, they may activate *Atoh7* expression, via Pax6 or Neurog2 binding, which would also displace nearby Rbpj-corepressor complexes. Conversely, during terminal differentiation, this relationship could be reversed, with Rbpj-corepressor binding at site R3 dislodging either Pax6 or Neurog2. Only the generation of single-cell genomic datasets can map the occupancy of particular enhancer binding site to the developmental status of cells, at distinct stages of retinogenesis.

Here we also provided some insight into *in vivo* context for our biochemical data, via direct comparison of Rbpj and Atoh7 protein expression patterns. Rbpj is well established as ubiquitously expressed, so co-localization with Atoh7 protein was already predicted. But, transcription factors that are coexpressed with their target genes typically activate transcription, not repress it. Indeed, Hes1, a known repressor of neurogenesis, displays mutually exclusive expression with βgal in *Atoh7*^*LacZ*/+^ eyes^26^. Yet, our transcriptional activity data clearly indicate that Rbpj repression of *Atoh7* is the major mode of regulation, which correlates with all previous genetic findings^25,29^. However, we also note that Rbpj does not appear uniformly expressed, with brighter anti-Rbpj labeling coinciding with Atoh7 expression. The significance of this observation remains unclear. Better understanding will require the determination of which Rbpj regulation activities at work during distinct stages of retinal cell development. Moreover, we must clarify whether Notch1/3 signaling invokes canonical pathway regulation, namely Notch-Rbpj-Maml binding to *Hes* gene promoters, which may in turn directly repress *Atoh7* transcription.

While highly speculative, we propose that lower levels of Rbpj protein expression are sufficient for binding to multiple CSL sites, which contributes to keeping the *Atoh7* locus open and primed (yet transcriptionally silent)^33^, until its rapid, pulsatile expression is needed. Higher levels of Rbpj protein may subsequently be required to also engage in the activities of Notch and co-repressor complexes, which act at different regulatory sequences, and possibly at different rates to shut down *Atoh7* transcription. Clearly additional transcription factors must simultaneously regulate *Atoh7* (positively or negatively) since brighter-labeled Rbpj cells are capable of Atoh7 co-expression. The integration of these multiple modes of regulation allows for more precise modulation of target gene mRNA levels, particularly during highly dynamic developmental processes.

## Experimental Methods

### Ethics Statement

All mice were housed and cared for in accordance with the guidelines provided by the National Institutes of Health, Bethesda, Maryland, and the Association for Research in Vision and Ophthalmology, and conducted with approval and oversight from the Cincinnati Children’s Hospital Research Foundation and UC Davis Institutional Animal Care and Use Committees.

### Animals

A *Rbpj*^*tm1Hon*^ conditional allele (termed *Rbpj*^CKO^) were maintained on a 129/SvJ background and genotyped as described^41^. α-Cre transgenic mice were maintained on a CD-1 background and genotyped as described^38^. Le-Cre mice were maintained on an FVB/N background and genotyped as described^40^. Z/EG lineage tracing mice (Tg(CAG-Bgeo/GFP)21Lb3/J) use the CMV enhancer/chicken actin promoter to constitutively express *lacZ*, which is replaced with *eGFP* expression upon Cre activation^39^. These mice were acquired from Jackson Labs (Stock Number 003920), maintained on a CD-1 background and genotyped as in^39^. Embryonic gestational age was determined by timed matings, with the date of the vaginal plug as E0.5.

The upstream, noncoding 2.4 Kb of mouse *Atoh7* genomic DNA (nucleotides −3032 to −503 containing the primary enhancer but lacking the TATAA box) was PCR amplified using primers with engineered XbaI and BglII restriction sites, cloned into the Xba I-Bam HI sites of the pBGnCherry vector^47^ in the normal transcriptional orientation, and verified by Sanger sequencing. The *Atoh7* fragment was PCR amplified (EXPAND Hi-Fidelity polyermase) from a previously subcloned 6.5 Kb mouse *Atoh7* genomic DNA template^3^, digested with BglII and XbaI and then purified. The pBGnCherry vector^47^ contains a minimal human β-globin promoter and monomeric Cherry red fluorescent protein reporter cassette (mCherry), as well as a synthetic amino terminal nuclear localization signal (MAPKKKRKVEDV) downstream of the BamHI site. There is no intrinsic activity of this vector in transgenic mice^47^. Linearized DNA was microinjected into CD-1 mouse pronuclei by the CHRF Transgenic Core Facility. F_0_ embryos were collected at E13.5 and screened for live mCherry fluorescence with a Leica MZ12 dissecting scope equipped with a Texas-Red filter. We harvested 4 Cherry +/44 embryos and each Cherry-positive embryonic head was cryoembedded, sectioned and analyzed using immunohistochemistry and confocal imaging (see below).

### Bioinformatics of Rbpj binding sites

Three kilobases of 5′ and three kilobases of 3′ noncoding genomic DNA from the mouse and human *Atoh7* genes (Gene IDs 53404 and 220202) were aligned using the MacVector Clustal W algorithm (v. 12). CNEs were identified in multiple vertebrate genomes using the UCSC genome browser MultiZ alignment and conservation features and mm10 genome assembly (http://genome.ucsc.edu). Putative CSL/Rbpj binding sites were identified using the TRANSFAC MATCH program with matrices M01111 (V$RBPJK_Q4) and M01112 (V$RBPJK_01). Previously defined Pax6 paired domain and E-box binding sites within the mouse *Atoh7* primary distal CNE are included for reference^8,10,11^.

### Immunohistochemistry

Embryonic heads were fixed in 4% paraformaldehyde/PBS for 1 hour at 4 °C, processed through a sucrose/PBS series, cryoembedded and sectioned at 10 μm. Immunohistochemistry using our lab protocol^2^ to label with chick anti-GFP (Abcam, 1:1000, AB13970), rat anti-Rbpj (CosmoBio, 1:100, SIM-2ZRBP-1), rabbit anti-Atoh7 (Novus Biologicals, 1:500, NBP1-88639), or goat anti-mCherry polyclonal (SICGEN, 1:500, AB0040-200) primary antibodies. Secondary antibodies were directly conjugated to Alexa Fluor 488 (Invitrogen, A21208), Alexa Fluor 594 (Jackson ImmunoResearch, 712-586-153), Alexa 594 (Invitrogen, A11058), Alexa Fluor 647 (Invitrogen, A21244), or Dylight 649 (Jackson ImmunoResearch, 711-495-152). Microscopic imaging used either a Zeiss Axioplan fluorescent microscope with a black and white camera, Apotome deconvolution device, and Axiovision (v. 7.0) software, or a Leica DM5500 microscope equipped with a SPEII solid state confocal and Leica LASAF software. All digital micrographs were electronically adjusted equivalently for brightness, contrast and pseudocoloring using Adobe Photoshop CS5 software.

### Western blotting

Pairs of lenses were harvested from E14.5 *Rbpj*^CKO/CKO^, Le-Cre;*Rbpj*^CKO/+^ and Le-Cre;*Rbpj*^CKO/CKO^ embryos and flash frozen. Twenty lenses (10 pairs) of the same genotype were pooled and lysed in RIPA buffer (50 mM Tris-HCl pH 8.0, 150 mM NaCl, 0.5% sodium deoxycholate, 0.1% SDS, 1% NP40) containing Complete protease inhibitors (Sigma 11697498001). Total protein concentrations were determined by Bradford assay (Biorad, 500-0006). NuPAGE 4–12% Bis-Tris gels (Invitrogen, NP0322BOX) were loaded with 20ug of total lens protein per gel lane, electrophoresed and transferred onto nitrocellulose membranes (Invitrogen, LC2000). Standard western blotting was performed, using rat anti-Rbpj (Cosmo Bio Co, 1:500 SIM-2ZRBP-2) or mouse anti-β-actin (Sigma, 1:3000, A1978) primary antibodies, followed by HRP-conjugated anti-rat IgG or mouse IgG secondary antibodies (Jackson Immunoresearch, Rat 112-035-175 1:5000, Mouse 315-035-003, 1:10,000). Blots were developed using a Supersignal West Pico Chemiluminescent substrate kit (Thermo Scientific, 34078), Kodak standard x-ray film and film developer.

### Electrophoretic Mobility Shift Assay (EMSA)

Single-stranded complementary oligonucleotides containing predicted CSL binding sites were labeled using a Biotin 3′ end DNA labeling kit (Thermo Scientific, 89818). Double-stranded DNA probes were made by annealing biotin-labeled complementary oligonucleotide pairs at room temperature for one hour. 0.5 μM purified mouse Rbpj protein (residues 53–474; Friedmann *et al*., 2008) and 1 nM of labeled oligonucleotide complexes, in the presence of 1.9 ng/μl poly[d(I-C)] (Sigma, 10108812001), were resolved on a 6% DNA retardation gels (Invitrogen, EC63652BOX) in 0.5 × TBE buffer and then transferred to nylon membranes. The LightShift Chemiluminescent EMSA kit assay (Thermo Scientific, 20148) was performed on the blots, which were developed using the Chemiluminescent Nucleic Acid Detection kit (Thermo Scientific, 89880), Kodak x-ray film and film developer.

### Isothermal titration calorimetry of CSL-DNA complexes

The production and purification of bacterially expressed Rbpj protein, residues 53–474, has been described^16,42^. Oligonucleotides from Integrated DNA Technologies (IDT) (Fig. 4A) were hydrated, purified, quantified and annealed as in^16^. All purified components were degassed, buffer matched and quantified as previously described^16^. A typical experiment was performed at 5 °C using a MicroCal VP-ITC microcalorimeter with the oligomeric duplex (~100 μM) in the syringe and Rbpj (~10 μM) in the cell and consisted of 40 injections of 7 μl each. Data analysis used the ORIGIN software and was fitted to a one-site binding model, with binding data representing the average of n = 3 experiments.

### Chromatin immunoprecipitation and Real-time PCR

ChIP was performed as described^10,76^ with several modifications. 30 E14.5 CD-1 pooled embryonic retinas were crosslinked with 1% formaldehyde and the reaction stopped by addition of 125 mM final concentration of glycine. Chromatin was sheered to 300–1000 bp size range with a Bioruptor UCD-200 sonicator + chiller (Diagenode), for 20 minutes at high power with 15 sec ON/30 sec OFF cycles. Either 3 μg rat anti-Rbpj antibody (Cosmo Bio Co, SIM-2ZRBP1) or rat IgG (Jackson ImmunoResearch, 012-000-003) were incubated with 40 μg sonicated chromatin overnight at 4 °C. Immune complexes were collected with Protein G agarose beads (Sigma, P7700), washed several times and eluted using 0.5 M NaHCO_3_, 1% SDS elution buffer. Pax6 ChIP was run in parallel from each retinal chromatin prep, by incubating 20 μg of sheared chromatin with 1 μg anti-Pax6 (Covance, PRB-278P), or rabbit IgG (Jackson ImmunoResearch, 011-000-003), coupled to Protein A sepharose beads (GE Healthcare, 17-0780-01). Input and immunoprecipitated chromatin samples were initially analyzed by performing 30 cycles of PCR amplification and agarose gel electrophoresis, then quantified by real time PCR, using Table S4 primers, fast SYBR Green master mixes and a StepOnePlus PCR system (Applied Biosystems, 4385612 and 4376600). A standard curve using serial dilutions of 1% input chromatin was used to calculate the percent input of each sample. The p-values were determined by ANOVA and a Bonferroni posthoc test (Rbpj) or a student’s unpaired, 2-tailed t-test (Pax6) with GraphPad Prism software (v6).

### Luciferase Assay

The upstream 2.6 Kb noncoding DNA from the mouse *Atoh7* locus was previously cloned into the pGL2 luciferase vector^10^. Rbpj binding site mutations were generated using PCR-based site directed mutagenesis^77^ and verified by Sanger Sequencing. Either 3.5 × 10^5^ HEK293T or 5 × 10^5^ AD12HER10 cells were plated per well of a 6-well tissue culture plate. After 48 hours (~60% confluency) cultures were transfected according to the Fugene6 (Promega, E2692) protocol with a 5:1 Fugene6 to DNA ratio, with the DNA constituting 500 ng of luciferase plasmid and 50 ng of Renilla control plasmid (pRL). In Notch ICD overexpression experiments, 100 ng of NICD1/pBK-CMV, 100 ng of NICD3/p3XFLAG-CMV-7TM, or a mixture of 50 ng of each plasmid were cotransfected with the luciferase and Renilla plasmids. Cells were washed in PBS, harvested 48 hours after transfection in 500 μL 1 × PLB (Promega) and cell pellets stored at −80 °C. Cell extracts were assayed in technical triplicate using the Dual Luciferase Assay System (Promega, E1980) on a Perkin Elmer Victor X5 workstation. Luciferase activity levels were normalized to the control Renilla activity, and p-values determined with GraphPad Prism (v6) software, using a two-tailed, unpaired t-test with equal standard deviation and assuming a Gaussian distribution.

### RT-PCR

Total RNA was extracted using the RNeasy micro kit (Qiagen, Cat No 74004) from 1 pair of dissected E13.5 retinas, or using Trizol (Invitrogen Cat No 15596026) for HER-10 cells. For embryonic retinal RNA 100 ng was reverse transcribed into cDNA using the iScript Synthesis kit and product protocol (BioRad, Cat No. 178891). For HER-10 cells, 6.5 μg of total RNA was first treated with 1U of 10 U/μL of DNase (Roche Cat No. 04716728001) by incubating at 37 °C × 35 min, 80 °C × 5 min, 90 °C × 3 min. Then 2 μg of treated RNA was used for cDNA synthesis, with Superscript III and manufacturer protocol (Invitrogen/ThermoFisher, Cat No. 18080093). Both experiments included a mock synthesis (lacking total RNA) performed in parallel. For embryonic retinas, 1 μL of cDNA was combined with individual primer sets (Supplemental Table S4) and Go-Taq polymerase (Promega, cat # M7122) for 35 cycles of PCR at 95 °C × 30 sec, 55 °C × 30 sec, 72 °C × 30 sec. PCR products were electrophoresed on a 2% TAE agarose gel. Alternatively, 1 μL HER-10 cDNA was combined with individual primer sets (Supplemental Table S4), 1 × PCR Buffer and dNTPs, 1 × Masteramp (Epicentre/Illumina, ME81210) and 1U of Taq polymerase (5U/μL Roche/Sigma, Cat No. 11146173001) for 35 cycles of PCR at 95 °C × 30 sec, 60 °C × 30 sec, 72 °C × 30 sec. PCR products were electrophoresed on a 1% TBE agarose gel.

### Data availability

All data generated or analyzed during this study are included within this published article and its Supplementary Information files.

## Electronic supplementary material

Supplemental Files
