# Supplementary material for: Rbpj direct regulation of Atoh7 transcription in the embryonic mouse retina
Source: Sci Rep. 2018 Jul 5;8:10195. doi: 10.1038/s41598-018-28420-y (PMC6033939; doi:10.1038/s41598-018-28420-y)
Supplement: Supplementary file 1 — Supplemental Files [file 41598_2018_28420_MOESM1_ESM.pdf]

Rbpj direct regulation of *Atoh7* transcription in the embryonic mouse retina

Joel B. Miesfeld[1#], Myung-soon Moon[1,2#], Amy N. Riesenberg[2] Ashley N. Contreras[3,4], Rhett A. Kovall[3] and Nadean L. Brown[1,2*]

[1]Department of Cell Biology & Human Anatomy, University of California Davis School of Medicine, One Shields Avenue, Davis, CA 95616

[2]Division of Developmental Biology, Cincinnati Children's Hospital Research Foundation, 3333 Burnet Avenue, Cincinnati, OH 45229

[3]Department of Molecular Genetics, Biochemistry and Microbiology, University of Cincinnati School of Medicine, Cincinnati, OH 45267

[4]Present address: Department of Biology, University of Cincinnati Blue Ash College, Cincinnati, OH 45236

#Co-First Authors

* Corresponding Author: Nadean L. Brown (nlbrown@ucdavis.edu)

Address: Department of Cell Biology & Human Anatomy, Room 4407, Tupper Hall University of California, Davis Davis, CA 95616

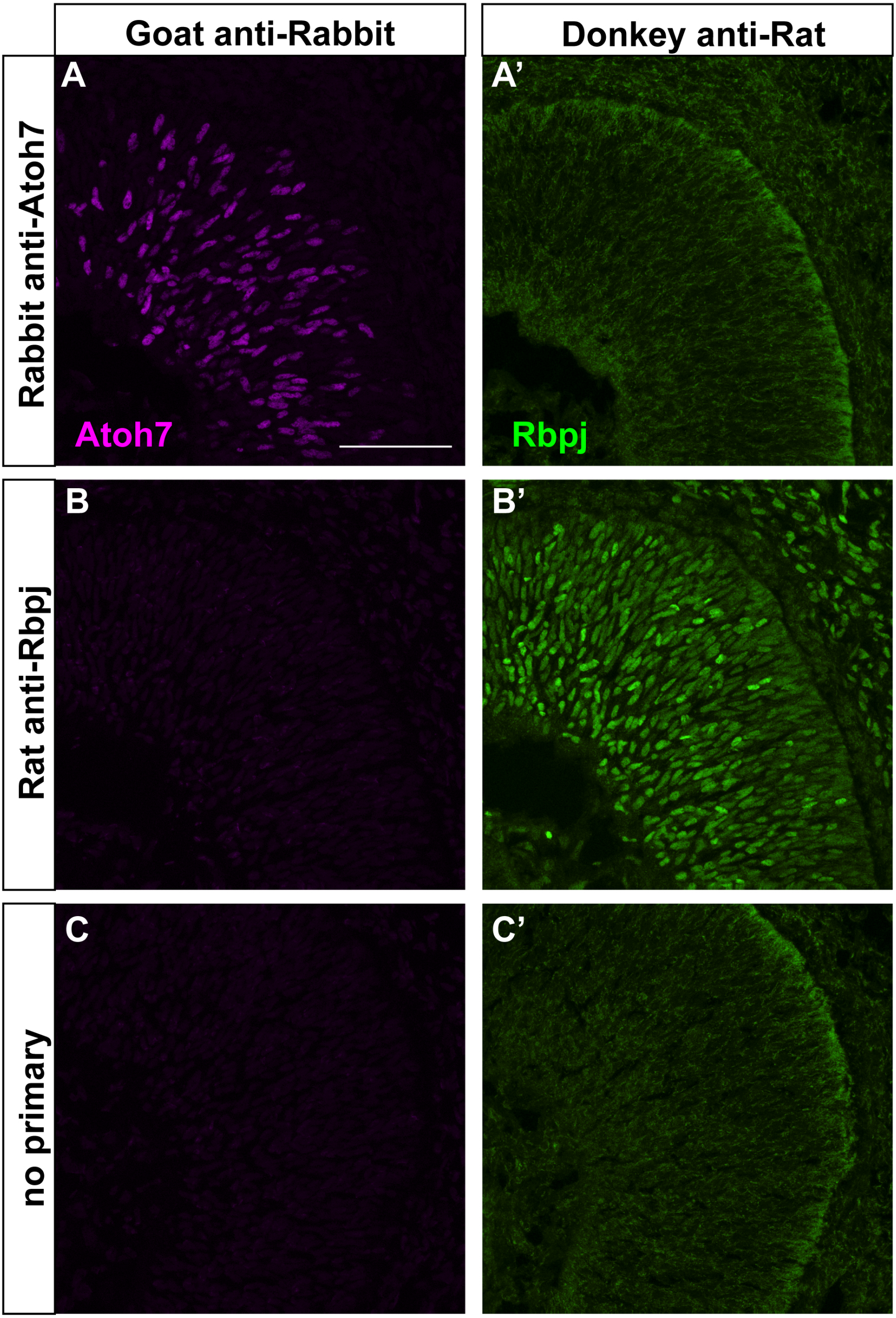

Supplemental Figure S1. Colocalization of Atoh7 and Rbpj proteins is not due to cross-species reactivity of secondary antibodies. A-C') E13.5 retinal cryosections were incubated sequentially with one primary antibody (indicated at left) and one secondary antibody (indicated at top), and processed as described in Methods. Panels in each column were imaged identically. Secondary antibodies were goat anti-rabbit IgG Alexa-647 and donkey anti-rat IgG Alexa-488, respectively. Section in C-C' received no primary antibody (blocking solution only). Bar = 50µm.

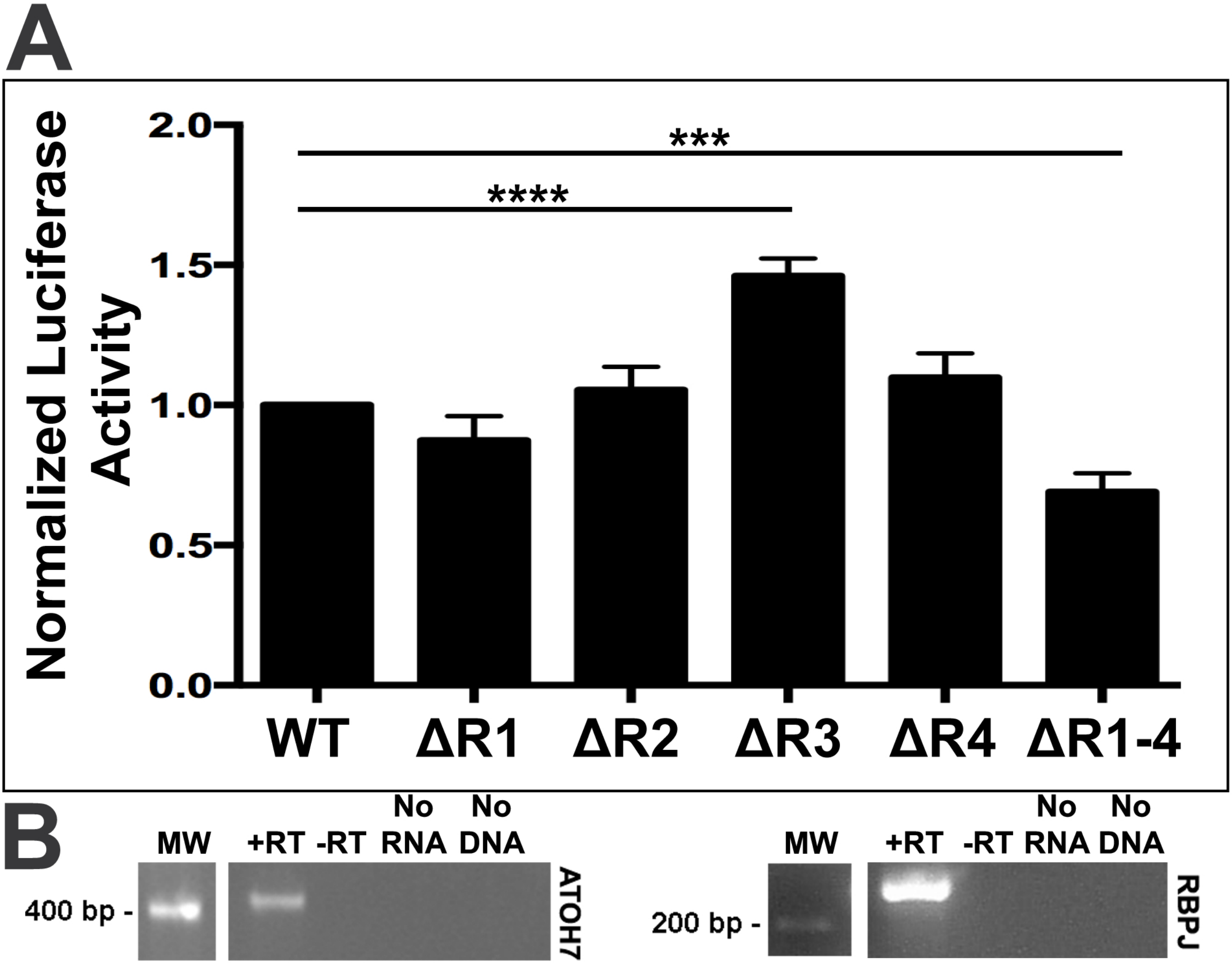

Supplemental Figure S2. Luciferase activity in Ad12HER10 retinal cells. A) Single and quadruple Rbpj binding site mutant luciferase assay comparison using a retinoblast cell line. The same outcomes were observed as in HEK293T cells, only ΔR3 site mutant derepressed Atoh7-luciferase activity, whereas simultaneous removal of all four CSL sites R1-4 reduced Atoh7-luciferase activity over wild type. $***p \leq 0.001$; $****p < 0.0001$; n = 9 biological replicates (each in technical triplicate). B) RT-PCR analysis of AD12Her10 cell mRNA showing endogenous RBPJ and ATOH7 expression. Uncropped gels provided in Suppl Figure S3.

Supplemental Figure S3 - Full size Western Blot exposures and RT-PCR gel images

Figure 2C Western

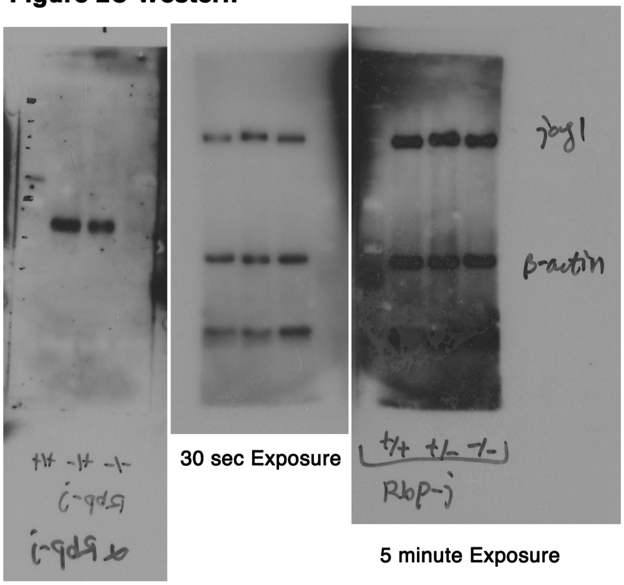

30 sec Exposure

5 minute Exposure

Anti-Rbpj Western
Long exposure

Anti beta-actin and anti-Jag1 colabel
reprobing of blot after stripping

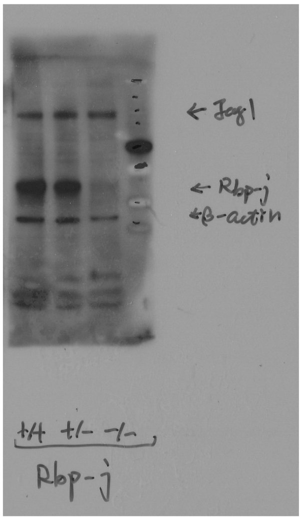

Independent Western blot with very short strip in between Rbpj and Actin/Jag1 antibody labeling

Figure 4A EMSA

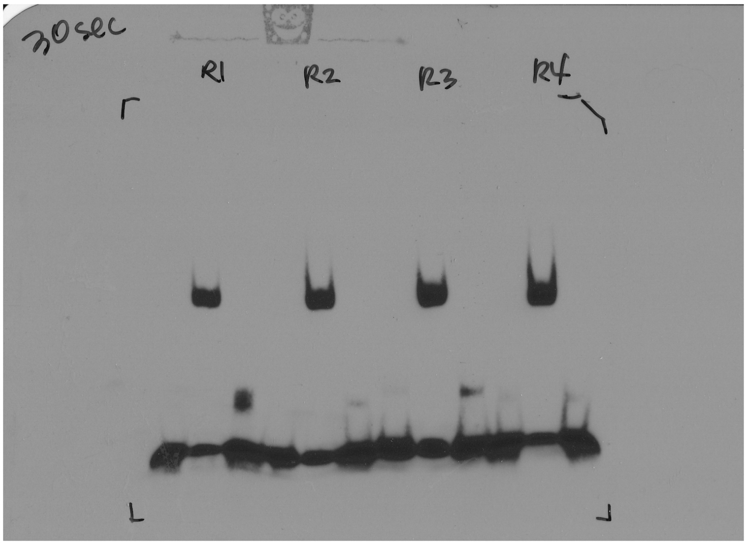

Figure 6C Full size gel images

pan-Fhl1			Fhl1B			Fhl1C			Spen		
+RT	-RT	no RNA	+RT	-RT	no RNA	+RT	-RT	no RNA	+RT	-RT	no RNA

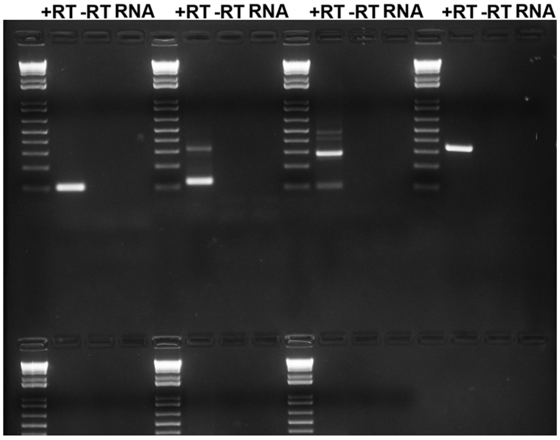

L3MBTL3			Kdm1A			Atoh7		
+RT	-RT	no RNA	+RT	-RT	no RNA	+RT	-RT	no RNA

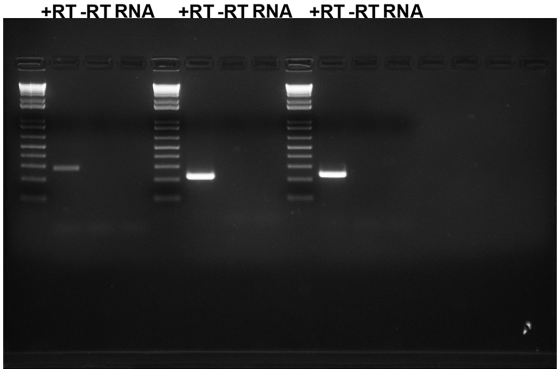

Supplemental Fig S2 Full size gel images

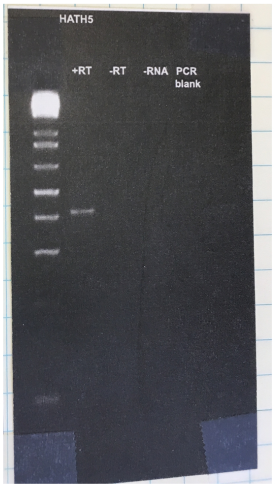

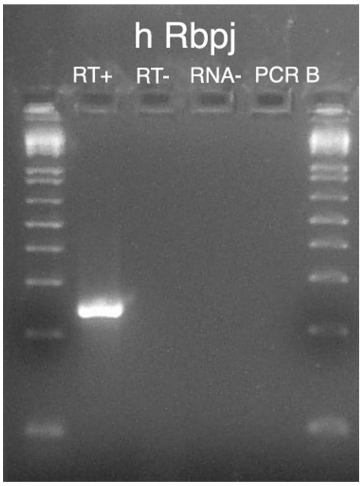

Supplemental Table S4 PCR primers used for ChIP or RT-PCR

ChIP qPCR primer sets for mouse *Atoh7* locus

Primer	Forward	Reverse	Amplicon	Start position
R1	TCCGTCATGAGTGTCACAGG	ACTAAAAGGGTGTCAGGGGG	137	-2280
R2	AGCCCAAAGAAAGCAGAC	CCATCACTCCTCGGTCCTAA	132	-1863
R3	CCAACATCTGTCGCTCTGAA	GCCCCGTCTTCCCATAAATA	126	-1700
R4	GACGAAGGCAATGATGTCAG	TCAAGGAGGGAACAGGTGGT	129	-539
NegControl	GGGTCTTAGGGGAGAAAACTCAATC	TACAAAACACAAGAGAAGTGGAGATA	114	+466
Neg-Pax6	ATCCTTCCCCTGACTGTGCCAC	CAAGATGCTTCCGCTTTTCTGG	208	+3026

RT-Primers Mouse

Primer	Forward	Reverse	Amplicon	NCBI gene ID
Pan *Fhl1**	GACTGCCGCAAGCCCATAA	CCAAGGGGTGAAGGCACTT	118	14199
*Fhl1B**	CTGCGTGGATTGCTACAAGA	CACACTGGGGACTTCCTAGC	126	14199
*Fhl1C**	TGCCATGAGACCAAGTTCG	GTAGTCGTGCCAGGATTGTC	101	14199
Spen	CCACGAACCTTCCGAAACATC	TCGATCTCCGCTGTAGAGAAC	340	56381
L3mbtl3	AGGGGTTCTCAGAGAGCCAG	TCCAAACAAATACAAGGCGGAG	282	237339
Kdm1A	GTGGTGTTATGCTTTGACCGT	GCTGCCAAAAATCCCTTTGAGA	228	99982
Atoh7	ACAAGAAGCTGTCCAAGTAC	AATGGCCCCGAGGCTTAGCTG	244	53404

*Primer sequences for these splice variants based on Figures 1 and S1 from Domenighetti et al., 2014

RT-Primers Human

Primer	Forward	Reverse	Amplicon	NCBI gene ID
ATOH7	TGGGTCTCCACTGTGAGCACTT	GCCGAAGTGCTCACAGTGGAG	439	220202
RBPJ	AACAAATGGAACGCGATGGTT	GAGATGACTTTTATCCGCTTGCT	241	3516

Supplemental Table S5: Calorimetric data for RBPJ binding to *Atoh7* DNA sites.

	Syringe	*Cell*	*K* (M^{-1})	K_d (μM)	*ΔG°* (kcal/mol)	*ΔH°* (kcal/mol)	*-TΔS°* (kcal/mol)
	Hes1 Consensus	RBPJ	1.1 ± 0.6 x 10^6	1.05	-7.6 ± 0.2	8.8 ± 1.2	16.4 ± 0.9
Atoh7 DNA	Site 1	RBPJ	8.3 ± 1.7 x 10^5	1.24	-7.5 ± 0.1	4.7 ± 0.2	12.2 ± 0.1
Atoh7 DNA	Site 2	RBPJ	3.4 ± 1.7 x10^5	3.64	-6.9 ± 0.3	3.7 ± 0.7	10.7 ± 0.4
Atoh7 DNA	Site 3	RBPJ	n/a	>10	n/a	n/a	n/a
Atoh7 DNA	Site 4	RBPJ	1.3 ± 0.4 x 10^6	0.81	-8.1 ± 0.4	5.1 ± 0.4	13.2 ± 0.4

All experiments were performed at 5°C, except for Site 3 (10°C). Values are the mean of at least three independent experiments and errors represent the standard deviations of multiple experiments. Weak binding was observed for Site 3, which only allowed for estimation of K_d. The Hes1 positive control DNA corresponds to the site –GTTACTGTGGGAAAGAAAG–.
